# Acute kidney injury in hematological patients treated with CAR-T cells: risk factors, clinical presentation and impact on outcomes

**DOI:** 10.1038/s41598-024-77720-z

**Published:** 2024-11-06

**Authors:** Elisa Russo, Massimiliano Gambella, Anna Maria Raiola, Elena Beltrametti, Valentina Zanetti, Giuseppe Chirco, Francesca Viazzi, Emanuele Angelucci, Pasquale Esposito

**Affiliations:** 1 Department of Internal Medicine and Medical Specialties (DIMI), University of Genova, Genova, Italy; 2 Unit of Nephrology, Dialysis and Transplantation, IRCCS Ospedale Policlinico San Martino, Genova, Italy; 3 Unit of Hematology and Cellular Therapy, IRCCS Ospedale Policlinico San Martino, Genova, Italy

**Keywords:** Acute kidney injury, Performance status, CAR-T cell therapy, Cytokine release syndrome, Disease-free survival, Mortality, Nephrology, Haematological cancer

## Abstract

Chimeric antigen receptor T-cell (CAR-T) therapy has revolutionized the treatment of hematologic malignancies, yet it carries significant risks, including acute kidney injury (AKI). In this study, we investigated the risk factors and clinical impact of AKI in patients undergoing CAR-T cell therapy. This retrospective study involved hematologic patients treated with CAR-T therapy. Clinical and laboratory data were collected, and clinical outcomes were monitored during follow-up after CAR-T infusion. AKI was defined according to KDIGO criteria. The outcome measures included early mortality, overall survival (OS), and disease-free survival (DFS). Among the 48 patients analyzed, 14 (29%) developed AKI, with a mean onset of 6 days after CAR-T infusion. The risk of AKI was associated with baseline performance status (OR 8.65, IC95% 6.2–12, *p* = 0.032) and the development of severe cytokine release syndrome post-therapy (OR 16.4 95%CI 1.9-138.5, *p* = 0.01). Patients with AKI more frequently required intensive care. Furthermore, severe AKI was independently associated with worse clinical outcomes, including reduced OS and DFS (HR 18.2, 95%CI 2.6–27.3, *p* = 0.003). Additionally, patients who developed AKI post-CAR-T therapy were more likely to progress to chronic kidney disease during follow-up. In conclusion, frail patients undergoing CAR-T therapy are at an increased risk of developing AKI, which can significantly affect both short- and long-term outcomes. Preventive strategies and early recognition of AKI are essential in these patients.

## Introduction

Chimeric antigen receptor T-cell (CAR-T) therapy represents an innovative approach for the treatment of hematological malignancies. CAR-T cells are genetically engineered immune cells, designed to recognize and bind specific proteins present on cancer cells^1^.

This treatment consists in collecting autologous T-cells, and reinfusing them into the patient, after genetic modification and in vitro activation/expansion.

These modifications induce the T-cells to express CARs on their surface, which, by binding tumor-specific antigens, trigger T-cell activation and subsequent cancer cell destruction^2^.

Current indications for CAR-T treatment include relapsed or refractory B-cell leukemia, lymphoma and multiple myeloma, potentially leading to disease remission, with increased overall and disease-free survival^3–5^. However, CAR-T therapy indications are still evolving, and growing evidence supports its application in a broader range of diseases, including non-neoplastic conditions such as autoimmune disorders^6^.

Despite its promise, CAR-T therapy is not without risks, and several adverse events have been reported. Potential complications include cytokine release syndrome (CRS), neurological issues like immune effector cell-associated neurotoxicity syndrome (ICANS), hematological and cardiac toxicity, and acute kidney injury (AKI)^7^. The most prevalent and significant complication is CRS, which affects a high proportion of patients undergoing CAR-T treatment^8^. CRS is a systemic inflammatory response triggered by the CAR-T cell-mediated rapid and massive release of pro-inflammatory cytokines, such as interleukin-6 (IL-6)^9^.

Clinically, CRS can manifest as mild symptoms such as fever and flu-like symptoms, or as more severe toxicities, including hypotension requiring vasopressors and hypoxia necessitating ventilatory support^10^. In severe cases, CRS may lead to life-threatening multiple organ dysfunction, including renal impairment.

AKI is a rather common complication in both pediatric and adult patients undergoing CAR-T therapy with an incidence ranging from 5 to 46% in different cohorts^11,12^.

In a systematic review including a population of 3376 pediatric and adult patients, Kanduri et al., described a pooled AKI incidence of 18.6% (17% in adult subjects)^13^.

Although the AKI incidence may vary among different populations, the occurrence of kidney damage in patients undergoing CAR-T treatment is not surprising. Indeed, these patients have multiple potential risk factors for AKI, including a history of cancer treated with multidrug regimens (including nephrotoxic agents), possible urinary tract obstruction related to hematological disease, and hyperinflammatory conditions such as CRS, which can impair kidney hemodynamics and perfusion^14^. However, although the relationship between AKI and CRS is well-established, the epidemiology of AKI in this clinical context, including specific individual risk factors for developing AKI, remains poorly defined.

The primary objectives of our study were to determine the incidence of AKI, describe associated clinical factors, and provide a more detailed characterization of how baseline conditions influence the risk of AKI in patients undergoing CAR-T therapy. Additionally, we assessed the impact of AKI on outcomes, including early and overall mortality and disease-free survival, acknowledging that not all AKI episodes are equivalent. The severity of AKI and the patients’ overall health status may significantly affect clinical outcomes.

## Methods

### Study design and data collection

We retrospectively analyzed patients undergoing CAR-T therapy for hematological indications at our center from 2020 to 2023.

We included all patients who underwent CAR-T treatment during the specified period, without applying specific exclusion criteria.

For these patients, we collected demographic data, comorbidity and hematological history, laboratory data, details of CAR-T infusion, and subsequent onset of complications and related treatments.

After the discharge, the follow-up visits were conducted based on clinical judgment.

The follow-up period concluded at the time of the first event (either disease recurrence or death) or at the last visit for patients still alive and free from disease (last observation on April 2024).

Comorbidities were assessed using the Charlson Comorbidity Index (CCI)^15^.

Presence of proteinuria was evaluated by dipstick urinalysis. Serum levels of interleukin-6 (IL-6), ferritin, and C-reactive protein (CRP) were measured using standard laboratory techniques.

The Ann Arbor staging system was used for the anatomic staging of disease^16^.

CRS was graded according to the American Society for Transplantation and Cellular Therapy (ASTCT) criteria, according to the degree of fever, hypoxia, and hypotension^17^.

ICANS was graded according to the severity of neurological symptoms, including cognitive dysfunction, level of consciousness, motor weakness, seizures, and cerebral edema, following the ASTCT guidelines^17^.

Patients’ functional status was assessed by application of The Eastern Cooperative Oncology Group (ECOG) performance status score^18^. Finally, the severity and prognosis of mantle cell lymphoma (MCL) and diffuse large B-cell lymphoma (DLBCL), were stratified by using the Mantle Cell Lymphoma International Prognostic Index (MIPI) and International Prognostic Index (IPI), respectively^19,20^.

The study protocol was approved by our institutional review board [Comitato Etico Regionale (CER) Liguria, Registration Number: 515/2020]. Due to the retrospective nature of the study, CER Liguria waived the need of obtaining informed consent. The study adhered to the principles outlined in the Helsinki Declaration.

### Definitions

Disease/tumor recurrence after CAR-T treatment was defined as any clinical or radiological evidence of disease, possibly confirmed by a confirmatory biopsy.

Ealy mortality was defined as mortality occurring for any causes within 30 days from CAR-T infusion.

Overall survival (OS) was defined as the interval from CAR-T cell infusion to death for any causes (uncensored) or the last follow-up date (censored).

Disease-free survival (DFS) was defined as the time from CAR-T treatment to the first event (either tumor recurrence or death, uncensored), or to the last follow-up date (censored).

Occurrence of AKI was evaluated during the hospitalization immediately following CAR-T infusion. AKI was defined using the Kidney Disease: Improving Global Outcomes (KDIGO) criteria, considering the increases in serum creatinine (sCr) levels compared to pre-CAR-T infusion values (set as baseline value)^21^. AKI was subsequently classified into three stages: stage 1 (sCr increase > 0.3 mg/dl and/or 1.5 to 1.9 times the baseline creatinine), stage 2 (2 to 2.9 times the baseline creatinine), and stage 3 (3 or more times the baseline creatinine).

Urine output was not considered for defining AKI due to limited available data.

Renal recovery was defined as a decrease in sCr to levels that no longer met the criteria for AKI when compared to baseline values.

Kidney function was assessed using the estimated glomerular filtration rate (eGFR), calculated with the Chronic Kidney Disease Epidemiology Collaboration (CKD-EPI) creatinine-based equation^22^. Chronic kidney disease (CKD) was defined as an eGFR < 60 mL/min.

### Endpoints and outcomes

The primary endpoint of the study was to determine the incidence of AKI in our cohort and to identify risk factors for its development. Additionally, we examined clinical and biochemical factors influencing patient outcomes, including length of hospital stay (LOS), early mortality, OS, and DFS.

### Statistical analysis

Characteristics of the patients were reported as mean with standard deviation (SD) or median with interquartile range (IQR) for continuous variables and as N (%) for the categorical ones; t-test and Pearson chi-square test or Fisher’s exact test were performed to compare patients with and without AKI. Univariate and multivariate logistic regression analyses were used to describe the relationship between all available variables at baseline and the development of AKI. Odds ratios and 95% confidence intervals were calculated by exponentiation of logistic regression coefficients.

Model fit was examined using the Hosmer-Lemeshow test, and *P* ≥ 0.05 was judged as a good model fit. Kaplan Meier survival analysis was used to assess DFS in patients with severe CRS (stage 4), and/or AKI. The survival curves between groups were compared by log-rank tests. The absence/presence of AKI stage 2/3 was tested as independent variable in multivariate Cox proportional hazard model analyses adjusted for the potential confounders.

All tests were two-sided and *p*-values < 0.05 were considered statistically significant. All statistical analyses were performed using Stata version 14.2 (Stata Corporation).

## Results

### Baseline general clinical characteristics

We analyzed a cohort of 48 patients (M/F: 29/19, mean age 61.4 ± 11.4 years) undergoing CAR-T therapy for diffuse large B-cell lymphoma (DLBCL) in 40 cases and mantle cell lymphoma (MCL) in 8 cases (Table 1).

**Table 1:** Baseline clinical-laboratory features in the whole cohort and based on AKI development.

	ALL	No AKI	AKI	*p*-value (No AKI vs. AKI)
N	48	34	14	
Age, years	61.4 ± 11.4	61.2 ± 10.4	61.8 ± 13.9	0.87
Sex M, n (%)	29 (60.4)	22 (64.7)	7 (50.0)	0.33
Hypertension, n (%)	18 (37.5)	12 (35.3)	6 (42.9)	0.62
Diabetes, n (%)	4 (8.3)	3 (8.8)	1 (7.1)	0.85
Charlson index median (IQR)	4 (2)	4 (2)	4.5 (3)	0.83
Hematological diagnosis, n (%)				
DLBCL	40 (83.3)	29 (85.3)	11 (78.7)	0.57
MCL	8 (16.7)	5 (14.7)	3 (21.4)	
Previous therapy lines, n (%)				1
2	38 (79.1)	27 (79.4)	11 (78.5)	
> 2	10 (20.9)	7 (20.6)	3 (21.5)	
ASCT, n (%)	12 (25)	11 (32.3)	1 (7.1)	0.07
Disease Stage 4, n (%)	24 (50)	16 (47)	8 (57)	0.70
IPI/MIPI High, n (%)	19 (39.6)	10 (29.4)	9 (64.3)	0.02
ECOG ≥ 3, n (%)	8 (18.7)	3 (8.8)	6 (42.9)	0.01
AKI stage				
1		–	8 (57.2)	
2		–	2 (21.4)	
3		–	4 (28.5)	
Laboratory				
sCr (mg/dl)	0.80 ± 0.24	0.80 ± 0.23	0.79 ± 0.27	0.93
eGFR (ml/min)	95.0 ± 19.2	96.0 ± 17.9	92.7 ± 22.8	0.59
Proteinuria (+ on dipstick)	7 (14.6)	5 (14.8)	2 (14.2)	1
LDH (U/L, normal range 135–225)	213 (128)	196 (102)	321 (447)	0.02
Albumin (g/dL)	3.3 ± 7.9	3.5 ± 6.7	2.97 ± 9.6	0.04
Mg (mg/dL)	2.1 ± 0.22	2.2 ± 0.25	2.03 ± 0.26	0.02
CRP (mg/L, normal range < 3)	26.3 ± 34.2	21.6 ± 32.1	37.7 ± 37.5	0.14
IL-6 (ng/L)	6.2 (9.4)	4.5 (8.0)	9.4 (18.4)	0.06
Ferritin (µg/L, normal range 24–336)	382 (780)	310 (534)	918 (1270)	0.10
Platelets (×10^9^/L)	116 (113)	136 (124)	86 (53)	0.02
Leukocytes (×10^9^/L)	1754 ± 1776	1859 ± 1859	1490 ± 1591	0.55
Lymphocytes (×10^9^/L)	224 ± 626	144 ± 305	424 ± 1073	0.19

*CAR-T* chimeric antigen receptor T-cell, *M* male, *NOS-DLBCL* diffuse large B-cell lymphoma, not otherwise specified, *MCL* mantle cell lymphoma, *ASCT* autologous stem cell transplant, *IPI* international prognostic index, *MIPI* mantle cell lymphoma international prognostic index, *ECOG* Eastern Cooperative Oncology Group, *LDH* lactate dehydrogenase, *CRP* C-reactive protein, *IL-6* interleukin-6, *sCr* serum creatinine.

Overall, 18 patients (37.5%) had a history of hypertension, and 4 patients (8.3%) had diabetes, with a median CCI of 4. Prior to CAR-T administration, all patients had received at least two lines of therapy, and 12 patients (25%) had also undergone autologous stem cell transplantation.

At the time of CAR-T treatment, 24 patients (50%) had Ann Arbor stage 4 disease (i.e.; extranodal disease), 19 patients (39.6%) had high IPI/MIPI scores, and 8 patients (16.7%) had an ECOG performance status score ≥ 3. Baseline sCr was 0.80 ± 0.24 mg/dL, with an eGFR of 95 ± 19.2 mL/min; three patients (6.2%) had pre-existing CKD.

Lastly, inflammatory markers, including CRP and ferritin levels, were elevated compared to normal laboratory ranges.

### AKI development and determinants

Using KDIGO criteria based on sCr changes, we identified AKI in 14 patients (29%), with a mean onset of 6 ± 3.6 days following CAR-T infusion. Among these patients, 8 (57.2%) developed AKI stage 1, 3 (21.4%) developed AKI stage 2, and 3 (21.4%) AKI stage 3.

On clinical basis, the etiology of AKI was primarily attributed to CAR-T therapy mainly because of the close temporal association between CAR-T infusion and the increase in serum creatinine.

We ruled out nephrotoxic causes, as all patients (both those who developed AKI and those who did not) underwent similar therapy immediately prior to CAR-T infusion. Additionally, renal ultrasounds were performed in all AKI patients, which showed no significant abnormalities, except in two patients who had urinary obstruction due to a lymphomatous tumor mass, a condition that was already present before CAR-T therapy and the onset of AKI.

A comparison of baseline characteristics between patients who developed AKI and those who did not revealed that the AKI group had worse prognostic indices and poorer performance status (Table 1), associated with lower serum albumin and magnesium levels. Baseline comorbidity and kidney function, such as prevalence of proteinuria, did not differ significantly between the groups.

Instead, patients who developed AKI had markedly higher levels of lactate dehydrogenase (LDH) and inflammatory markers, including IL-6, as well as lower platelet counts.

Multivariate logistic regression analysis, adjusted for general characteristics and disease stage, demonstrated that a high baseline ECOG performance status score was independently associated with an increased risk of developing AKI (Table 2). The multivariate model was significant and fitted well (Model χ^2^ test: *P* < 0.01, Hosmer-Lemeshow test: *P* = 0.22).

**Table 2:** Baseline clinical-laboratory features and AKI risk.

	Univariate			Multivariate		
	OR	95%CI	p	OR	95%CI	p
Age, years	1.00	0.95–1.06	0.86	0.98	0.98–1.08	0.73
Sex, male	0.54	0.15–1.93	0.35			
LDH, U/L	2.63	0.61–11.30	0.19			
IPI/MIPI high	4.32	1.15–16.15	0.03			
Disease stage 4	1.30	0.34–4.94	0.70	0.64	0.13–3.14	0.58
GFR, mL/min/1.73 m^2^	0.98	0.95–1.02	0.45	0.95	0.89–1.02	0.20
ECOG ≥ 3	7.75	1.58–37.97	0.01	10.3	1.59–66.41	0.01

Model χ^2^ test: *P* < 0.01, Hosmer-Lemeshow test: *P* = 0.22.

*CAR-T* chimeric antigen receptor T-cell, *M* male, *NOS-DLBCL* diffuse large B-cell lymphoma, not otherwise specified, *MCL* mantle cell lymphoma, *ASCT* autologous stem cell transplant, *IPI* international prognostic index, *MIPI* mantle cell lymphoma international prognostic index, *ECOG* Eastern Cooperative Oncology Group, *LDH* lactate dehydrogenase, *CRP* C-reactive protein, *IL-6* interleukin-6, *sCr* serum creatinine.

*IPI/MIPI score were excluded by multivariate analysis because of collinearity.

### CAR-T cell infusion management and complications

Before CAR-T infusion, 27 patients (56.2%) received a three-day lymphodepleting regimen consisting of fludarabine 25 mg/m^2^ and cyclophosphamide 250 mg/m^2^ before tisagenlecleucel infusion, while the remaining 21 patients received fludarabine 30 mg/m^2^ and cyclophosphamide 500 mg/m^2^ in advance of axicabtagene or brexucabtagene infusion, in accordance with the manufacturer indications (Table 3).

**Table 3:** CAR-T therapy schedule, post-infusion complications and management in the whole cohort and based on AKI development.

	ALL	No AKI	AKI	*p*-value (No AKI vs. AKI)
N	48	34	14	
CAR-T therapy				
Lymphodepletion regimen, n (%)				
Fluda 25 Cy 250	27 (56.2)	18 (52.9)	9 (64.3)	0.42
Fluda 30 Cy 500	21 (43.8)	16 (47.1)	5 (35.7)	
CAR-T product, n (%)				0.40
Tisagenlecleucel	26 (54.2)	17 (50)	9 (64.3)	
Brexucabtagene autolocel	8 (16.6)	5 (14.7)	3 (21.4)	
Axicabtagene ciloleucel	14 (29.2)	12 (35.3)	2 (14.3)	
Post-infusion complications				
CRS, n (%)	41 (85.4)	27 (79.4)	14 (100.0)	0.066
Severe CRS (3 + 4), n (%)	9 (21.9)	2 (7.4)	7 (50)	0.0026
CRS duration, days	6.5 ± 3.9	5.0 ± 1.5	9.6 ± 5.3	0.001
ICANS, n (%)	14 (29.2)	8 (23.5)	6 (42.9)	0.18
ICU admission, n (%)	13 (27.1)	5 (14.7)	8 (57.1)	0.003
Vasoconstrictor support, n (%)	6 (12.5)	0 (0.0)	6 (42.9)	< 0.001
IMV, n (%)	8 (16.7)	1 (2.9)	7 (50.0)	< 0.001
Peak sCr (mg/dl)	1.02 ± 0.38	0.89 ± 0.25	1.33 ± 0.46	< 0.001
Peak IL-6, (ng/L)	678 (3112)	159 (1159)	3192 (3539)	0.005
Peak CRP, (mg/L)	85 (114)	77 (86)	140 (82)	0.006
Peak Ferritin, (µg/L)	730 (1657)	560 (760)	3368 (28425)	< 0.001
Pharmacological treatment				
Tocilizumab, n (%)	34 (70.8)	22 (64.7)	12 (85.7)	0.18
Corticosteroids, n (%)	23 (47.9)	12 (35.3)	11 (78.5)	0.01
Anakinra, n (%)	19 (39.6)	8 (23.5)	11 (78.6)	< 0.001

*CAR-T* chimeric antigen receptor T-cell, *Fluda* fludarabine, *Cy* cyclophosphamide, *CRS* cytokine release syndrome, *ICANS* immune effector cell-associated neurotoxicity syndrome, *ICU* intensive care unit, *IMV* invasive mechanical ventilation, *IL-6* interleukin-6, *CRP* C-reactive protein, *sCr* serum creatinine, *AKI* acute kidney injury.

The most commonly administered CAR-T product was tisagenlecleucel, followed by axicabtagene ciloleucel and brexucabtagene autoleucel, with no differences between AKI vs. no AKI patients.

Following CAR-T infusion, 41 patients (85.1%) developed CRS of any grade, with 9 patients (21.9%) experiencing severe CRS grade 3 or 4. The mean duration of CRS was 6.5 ± 3.9 days. Additionally, 14 patients (29.2%) developed ICANS, and 13 patients (27.1%) required admission to the intensive care unit (ICU).

When comparing patients based on the development of AKI, we observed a higher incidence and a longer duration of CRS in the AKI group. Notably, all patients who developed AKI also had concurrent CRS, while none of the patients without CRS developed AKI. Furthermore, patients with AKI experienced more severe forms of CRS stage 3 and 4 compared to those without AKI.

Multivariate logistic regression analysis confirmed that severe CRS increased the risk of AKI by 16 times, independently by age, gender and disease stage (OR 16.4 95%CI 1.9-138.5, *p* = 0.01, Table 4).

**Table 4:** Post CAR-T cell infusion features and AKI risk.

	Univariate			Multivariate		
	OR	95%CI	p	OR	95%CI	p
Age, years	1.00	0.95–1.06	0.86	0.9	0.9-1.0	0.19
Sex, male	0.54	0.15–1.93	0.35	1.6	0.3–9.1	0.58
IPI/MIPI high	3.0	1.3–7.1	0.01	3.5	1.1–10.7	0.03
CRS grade ≥ 3	16.0	2.7–94.1	< 0.01	16.4	1.9-138.5	0.01

Hosmer-Lemeshow test: *P* = 0.53.

*CAR-T* chimeric antigen receptor T-cell, *IPI* international prognostic index, *MIPI* mantle cell lymphoma international prognostic index, *CRS* cytokines release syndrome.

Laboratory tests revealed that patients with AKI following CAR-T treatment had significantly elevated inflammatory markers compared to those without AKI. Additionally, AKI patients were more frequently admitted to the ICU and required hemodynamic and ventilatory support.

Then, evaluating AKI patients according to CRS severity, we observed that while there were not significant differences in IL-6 elevation [peak IL-6 in CRS stage 3–4 patients 4575(4500) vs. 2939 (2239) ng/ml in CRS 1–2 patients, *p* = 0.6], patients developing AKI in concomitance of severe CRS stage 3–4 had a significantly high need of intensive care support [seven (100%) required ICU admission and six (85%) vasopressor support].

Finally, while the use of tocilizumab was similar between the patients developing AKI and those without AKI, corticosteroids and anakinra were more commonly used in AKI patients.

### AKI management and time-course

For each patient who developed AKI, we provided guidance on optimizing volume status, closely monitoring electrolyte levels and acid-base balance, and avoiding nephrotoxic drugs. All these measures were implemented on an individual patient basis, guided by clinical judgment, without a standardized protocol. Moreover, among the fourteen patients who developed AKI, four (28.5%) required kidney replacement therapy (KRT), including one patient with AKI stage 2 and three with AKI stage 3. All the patients undergoing KRT had associated CRS stage 4.

The primary indications for KRT were the management of hyperinflammatory syndrome in three patients and the treatment of kidney failure in one patient who developed anuria.

All patients requiring KRT underwent continuous veno-venous hemodiafiltration.

Overall, in the AKI group, two patients (14%) died with unrecovered AKI, while the remaining twelve patients recovered kidney function within a median of 4 days (range: 4–10 days), without substantial differences in patients developing AKI in association of more severe CRS stage 3–4 [4 (5) days] compared with patients with CRS stage 1–2 [4 (6.2) days, *p* = 0.9].

Finally, two patients experienced a second episode of AKI during their hospital stay.

### Outcomes: determinants and impact of AKI

In terms of clinical outcomes, the mean LOS following CAR-T infusion was 25.6 ± 17.6 days, with significantly longer stays observed in patients who developed AKI (Table 5). Early mortality occurred in three patients (6.2%), all included in the AKI group (*p* = 0.02 vs. No AKI).

**Table 5:** Short and long-term outcomes in the whole cohort and based on AKI development.

	ALL	No AKI	AKI	*p*-value (No AKI vs. AKI)
N	48	34	14	
LOS, days	25.6 ± 17.6	21.7 ± 6.1	35.3 ± 29.7	0.013
Early mortality, n (%)	3 (6.2)	0 (0.0)	3 (28.6)	0.02
OS, days	210 (6-489) Range 6-1184	295 (138–516) Range 54-1184	70 (31–166) Range 6-915	< 0.001
Disease-free patients, n%	20 (41.7)	16 (47.1)	6 (29.6)	0.2
DFS, days	175 (58–389) Range 6-1184	249.5 (67–479)	65 (31–158)	0.003
Last follow-up, n patients*	45	34	11	
CKD, n (%)	4 (8.8)	1 (2.9)	3 (27.2)	0.039
De novo CKD	2	0	2	

*AKI* acute kidney injury, *LOS* length of hospital stay, *OS* overall survival, *DFS* disease-free survival, *CKD* chronic kidney disease.

*Patients with early mortality were excluded from this analysis.

Overall, at a median follow-up of 175 days (IQR 63–389) 26 patients had disease recurrence and 18 patients had died [8 patients (57%) in the AKI group, and 10 patients (29%) in the No-AKI group, *p* = 0.1]. Disease progression (88.8%, *n* = 16) was the most common cause of death.

Moreover, at the follow-up end, twenty patients (41.7%) were disease-free, with no significant difference between the AKI and non-AKI groups.

**Fig. 1:**
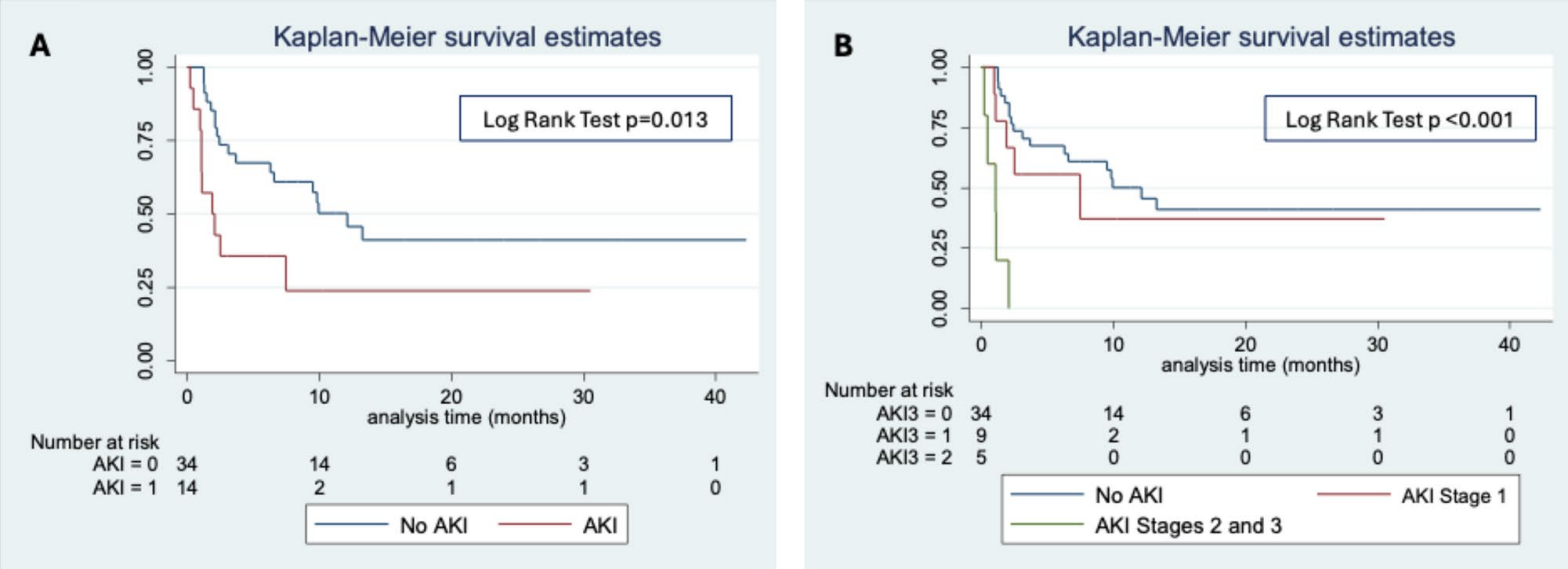
Kaplan Maier analysis of disease-free survival on the basis of (**A**) the presence of AKI and (**B**) the stages of AKI.

**Fig. 2:**
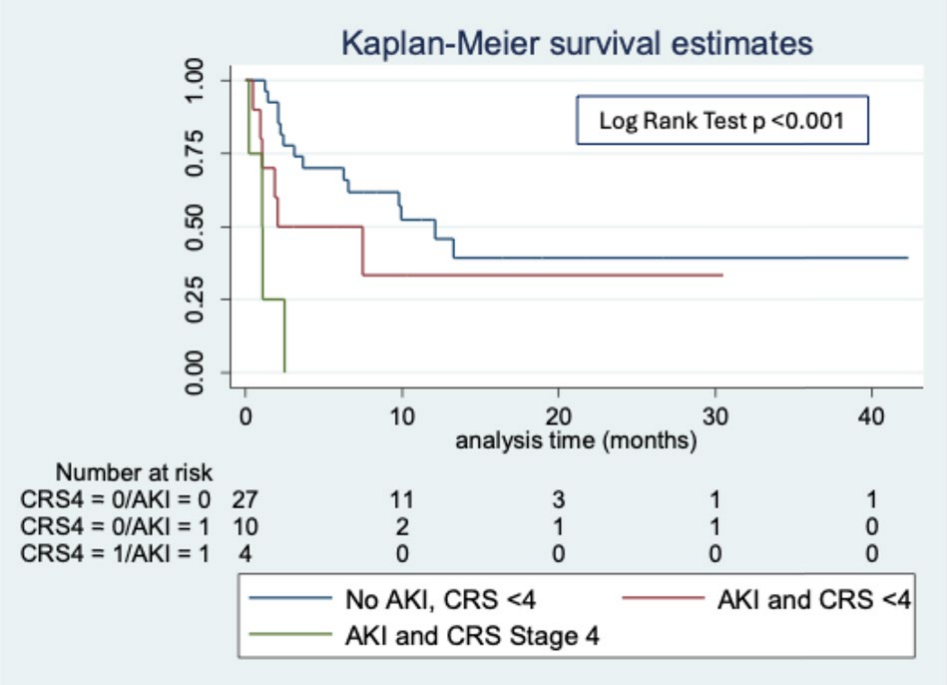
Kaplan Maier analysis of disease-free survival on the basis of the presence of AKI and CRS stage 4.

Instead, OS and DFS was significantly shorter in patients who developed AKI compared to those who did not. Kaplan-Meier analysis confirmed that patients with AKI had a lower DFS, which further decreased with the severity of AKI, particularly in those with AKI stage 2–3 (Fig. 1A,B). When analyzing the combined impact of AKI and CRS, patients with CRS complicated by AKI had a lower DFS compared to those who developed CRS without AKI (Fig. 2).

Multivariate Cox regression analysis identified age, male sex, high tumor burden, CRS stage 4, and severe AKI (HR = 18.2, 95%CI 2.6–27.3, *p* = 0.003) as independent risk factors for events (Table 6).

Finally, after excluding patients who experienced early mortality, a significantly higher number of those who developed AKI post-CAR-T infusion were diagnosed with CKD at the last follow-up visit.

**Table 6:** Cox regression analysis testing the factors affecting DFS after CAR-T treatment.

	Univariate			Multivariate		
	HR	95%CI	p	HR	95%CI	p
Age, years	1.02	0.98–1.06	0.290	1.01	0.94–1.07	0.846
Sex, male	1.49	0.68–3.25	0.316	4.85	1.48–15.90	0.009
No AKI	REF.					
AKI stage 1	1.51	0.56–4.11	0.412			
AKI stage 2	10.37	2.15–49.95	0.004			
AKI stage 3	28.65	5.38–35.26	< 0.001			
AKI, stage 2 and 3	14.16	4.44–45.17	< 0.001	18.2	2.6–27.3	0.003
Disease stage 4	4.69	1.59–13.84	0.005	4.29	1.28–14.43	0.019
ECOG ≥ 3	4.55	1.89–10.91	0.001	1.15	0.22–6.03	0.865
CRS 4	7.23	2.24–23.31	0.001	7.65	1.15–5.10	0.035

*DFS* disease-free survival, *CAR-T* chimeric antigen receptor T-cell, *AKI* acute kidney injury, *ECOG* Eastern Cooperative Oncology Group, *CRS* cytokine release syndrome.

## Discussion

In this study, we aimed to analyze the epidemiology, presentation, risk factors, and clinical correlates of AKI following CAR-T cell therapy in patients with hematological malignancies. We observed an overall AKI incidence of approximately 30% in our cohort. While this finding aligns with some previous reports, the literature reveals a wide range of reported AKI incidence, likely due to the varying criteria used for AKI diagnosis across studies. Notably, many studies lack a standardized AKI definition, and even those applying KDIGO criteria show variability in the timeframe for AKI diagnosis. For instance, Gupta et al.^23^ and Farooqui et al.^24^, assessed AKI occurrence within the first 30 days post-CAR-T therapy, reporting an incidence of approximately 20%. In contrast, Gutgarts et al. documented a 30% incidence (14/46 patients) within the first 100 days post-CAR-T therapy^25^.

Given the complexity of patients with hematological malignancies and their multiple comorbidities, attributing an AKI episode occurring long after therapy solely to CAR-T treatment remains challenging. To address this, we strictly applied KDIGO criteria, focusing on AKI occurring immediately after CAR-T cell administration. So, in our population, the mean time from CAR-T infusion to AKI diagnosis was 6 days. These different diagnostic approaches may explain some of the observed discrepancies in clinical presentation and the impact of AKI on outcomes across different populations.

Additionally, our study explored baseline risk factors for AKI development, an important yet under investigated area. Previous studies have predominantly focused on the relationship between AKI and post-CAR-T infusion complications, particularly CRS. This is crucial as it may provide insights into the pathogenesis of AKI, which is thought to be an expression of organic and functional alterations secondary to hyperinflammatory syndrome. However, it may be more informative to determine if the risk of developing AKI after CAR-T cell infusion can be correlated with baseline clinical, instrumental, or biochemical factors.

In this view, recently, Leon-Roman et al. described male sex and baseline eGFR as predictors of AKI in a cohort of 115 patients including 12% with baseline CKD^26^.

Interestingly, our data do not confirm the relationship between pre-treatment GFR and AKI risk, probably because of the different composition of our patient cohort, primarily consisting of patients with normal baseline renal function. On the other hand, the association between baseline kidney function and AKI in CAR-T patients remains debated. For instance, Wood et al., in a retrospective study of 166 patients, including 10% with baseline CKD, found no significant differences in AKI incidence or survival outcomes between patients with and without renal impairment^27^.

In our analysis, patients who developed AKI had worse baseline prognostic indices, poorer performance status, and lower levels of albumin and magnesium. Additionally, they exhibited higher levels of LDH, IL-6, and ferritin, along with reduced platelet counts compared to those without AKI. Multivariate analysis confirmed that a higher pre-treatment ECOG performance status was an independent risk factor for the subsequent development of AKI. These findings suggest that patients with both clinical^28^ and biochemical markers of frailty—such as hypoalbuminemia and hypomagnesemia—may be at elevated risk for AKI. These observations are particularly significant, as these markers are easy to evaluate, and recognizing risk factors early can help in planning preventive strategies and evaluating candidates for CAR-T therapy.

CRS is the most significant complication of CAR-T treatment in terms of incidence (85.4% in our cohort) and potential harm. CRS, associated with high morbidity and mortality, can affect multiple organs, including the kidneys.

The mechanisms linking CRS to kidney function impairment are complex, potentially involving both hemodynamic alterations and direct toxic effects^9,29^. Elevated cytokine levels can lead to kidney hypoperfusion through vasodilation, reduced cardiac output, and increased vascular permeability, which may result in intravascular volume depletion. These changes can alter renal hemodynamics, leading to functional damage and ultimately AKI.

Moreover, the high cytokine burden may also exert direct toxic effects on tubular cells and promote intrarenal inflammation, further contributing to kidney injury^14^.

Thus, it is not surprising that previous studies analyzing AKI occurrence after CAR-T treatment have found a significant correlation with CRS^23,30^.

Our findings align with this data, showing that CRS was strongly and independently associated with AKI. AKI was more prevalent in patients with CRS, who exhibited a marked increase in inflammatory markers.

Notably, AKI predominantly occurred in patients with severe CRS, who also presented a more complicated clinical course, including a higher need for intensive care.

Furthermore, all patients requiring KRT had CRS stage 4, with the management of CRS being the primary indication for initiating KRT.

In terms of treatment strategies, AKI patients more often required intensive care with vasoactive and respiratory support. While tocilizumab, the first-line treatment for CRS, was used equally between AKI and non-AKI patients, drugs such as corticosteroids and anakinra, indicated for refractory cases, were more often prescribed in AKI patients^31,32^. These findings support the concept of AKI development as a consequence of the severe inflammatory response following CAR-T treatment, necessitating a multi-target, intensive approach and longer hospitalization^33^.

Intriguingly, it has been shown that damaged kidney cells during AKI can themselves become sources of inflammatory molecules^34^. Therefore, it cannot be excluded that the concurrent development of AKI in patients with CRS may sustain and amplify inflammatory processes.

Monitoring the time course of AKI revealed that, consistent with previous studies, many cases were transient, with recovery occurring in a median of 4 days^26^. However, AKI significantly impacted patient outcomes. So, all three patients with early mortality experienced AKI, and two of them still had AKI at the time of death. Furthermore, while there were no significant differences in the absolute number of disease-free patients between those experiencing AKI and those who did not, the difference became evident when considering OS and DFS, which were significantly lower in AKI patients.

Multivariate analysis of clinical factors associated with outcomes showed that male sex, advanced hematological disease stage, CRS stage 4, and severe AKI were all independently and inversely associated with DFS.

The significant impact of AKI, independent of CRS, is a novel finding from our study, as previous research did not clearly demonstrate the detrimental effect of AKI on the prognosis of CAR-T therapy patients^25,30,35^. This disparity may be partly attributed to the different size, composition and settings of cohorts analyzed, and heterogeneous AKI diagnostic criteria adopted in various studies.

Furthermore, the choice of different outcomes may influence the results. In contrast to previous studies, which mostly evaluated overall mortality at different time points (30 or 60 days)^23^, we also analyzed the occurrence of adverse events, such as hematological disease recurrence, and evaluated DFS, a well-established outcome measure in cancer patients.

Finally, our results suggest that a more detailed analysis of AKI might be necessary to uncover its clinical effects. While previous studies examined the effects of overall AKI, we demonstrate that only the most severe forms of AKI impact patient prognosis.

An additional finding of our study is that patients who experienced AKI after CAR-T therapy had an increased risk of presenting CKD during follow-up, even if they initially recovered kidney function. The relationship between AKI and CKD, already noted in patients treated with CAR-T, may represent a complex problem, as CKD development can complicate both the general condition and subsequent hematological management, including access to further therapy lines and, for selected cases, allogeneic stem cell transplantation^36^.

Our study has several limitations, primarily due to its retrospective design and relatively small sample size, which restrict our ability to establish clear causal relationships between clinical factors, laboratory parameters, and outcomes.

Additionally, because of the study’s design, no specific timepoints for AKI diagnosis and follow-up evaluations were predefined. As a result, temporal correlations between the onset of complications, treatments, and clinical outcomes could not be clearly recorded.

Furthermore, we did not adopt standardized protocols for AKI management and treatment, making it difficult to assess the individual effects of specific interventions on patient outcomes. For instance, anakinra was used to treat CRS in both patients with and without AKI, complicating the evaluation of its specific impact.

Another limitation is the lack of precise urine output evaluation for defining AKI, which is a significant limitation not only of our study but also of much of the existing literature on AKI epidemiology^37^.

Finally, while in our cohort the attribution of AKI to CAR-T therapy appears clinically plausible, it cannot be regarded as certain.

In conclusion, our data show that among patients eligible for CAR-T therapy, those who are more fragile have a higher risk of AKI, which is associated with the post-treatment inflammatory response and, at least in its most severe forms, represents a significant complication that impacts short-term clinical management and prognosis. It may also have long-term effects, increasing the risk of developing CKD.

These considerations highlight the importance of closely monitoring kidney function in patients undergoing CAR-T therapy, as it may be a critical factor in their overall prognosis. Further research, preferably prospective, is needed to clarify additional factors influencing AKI risk, define preventive strategies, and develop approaches to mitigate this complication in CAR-T-treated patients, potentially incorporating biomarkers for detecting preclinical kidney injury.

## Data Availability

The datasets used and/or analyzed during the current study are available from the corresponding author on reasonable request.
